# Amelioration of polycystic ovarian morphology by Tokishakuyakusan in a PCOS rat model: association with bone morphogenetic protein 4

**DOI:** 10.3389/fendo.2025.1649124

**Published:** 2026-01-09

**Authors:** Mako Ueda, Satoko Osuka, Atsushi Yabuki, Natsuki Miyake, Naoki Fujitsuka, Miwa Nahata, Yohei Tokita, Jiali Ruan, Bayasula, Takehiko Takeda, Tomomi Seki, Reina Sonehara, Ayako Muraoka, Tomoko Nakamura, Hiroaki Kajiyama

**Affiliations:** 1 Department of Obstetrics and Gynecology, Nagoya University Graduate School of Medicine, Nagoya, Japan; 2 Department of Obstetrics and Gynecology, Aichi Medical University School of Medicine, Nagakute, Japan; 3 TSUMURA Kampo Research Laboratories, TSUMURA & CO., Ibaraki, Japan; 4 Research and Development Division, TSUMURA & CO., Tokyo, Japan

**Keywords:** bone morphogenetic protein, Kampo medicine, polycystic ovary syndrome, progesterone, Tokishakuyakusan

## Abstract

**Background:**

Polycystic ovary syndrome (PCOS) is a common cause of irregular menstrual cycles and infertility. Current treatments primarily involve ovulation induction and sex steroid hormone therapy. Tokishakuyakusan (TSS) is a traditional Japanese medicine used for reproductive disorders. Bone morphogenetic protein 4 (BMP4), a regulator of follicular growth and steroidogenesis, may contribute to PCOS pathophysiology. This study aimed to investigate the effects of TSS on ovarian morphology, gene expression profiles, and steroidogenesis in a PCOS rat model.

**Methods:**

A Wistar rat model of PCOS was generated through prenatal dihydrotestosterone (DHT) exposure. Model rats were fed either a normal diet (DHT group) or a 3% TSS-supplemented diet (DHT+TSS group). Vehicle-treated control rats received a normal diet (vehicle group). Estrous cyclicity and ovarian histology were evaluated. Ovarian gene expression profiling and Western blot analyses were performed. Primary granulosa cells (GCs) isolated from healthy and model rats were treated with human follicle-stimulating hormone (FSH) and TSS to assess underlying mechanisms.

**Results:**

PCOS-like phenotypes, including irregular estrous cycles and polycystic ovaries with atretic cyst-like follicles, were observed in the DHT group. Compared with the DHT group, the DHT+TSS group showed a reduced number of atretic cyst-like follicles and improved estrous cyclicity. Ovarian gene expression profiling revealed lower *Bmp4* and *inhibin-βa* (*Inhba*) expression in the DHT+TSS group than in the DHT group. Consistent with these findings, BMP4 and inhibin βA protein levels were significantly decreased in the DHT+TSS group. In GCs from model rats, TSS treatment significantly reduced *Bmp4* and *Inhba* expression and enhanced FSH-induced *steroidogenic acute regulatory protein* (*Star*) expression and progesterone production.

**Conclusion:**

TSS ameliorated PCOS-like ovarian histopathology in prenatally DHT-treated rats and enhanced progesterone production by upregulating *Star* expression in GCs, accompanied by reduced BMP4 expression. These findings suggest that TSS may improve irregular estrous cycles and ovarian morphology in PCOS through the regulation of BMP4 signaling.

## 1 Introduction

Polycystic ovary syndrome (PCOS) is a common endocrine disorder associated with irregular menstrual cycles and infertility, affecting 10%–13% of women worldwide (1–3). PCOS is characterized by hyperandrogenemia, abnormal ovarian morphology, elevated anti-Müllerian hormone levels, and ovulatory dysfunction. This condition is further associated with increased risks of pregnancy-related complications and endometrial cancer. It is also closely linked to glucose intolerance, insulin resistance-associated cardiovascular disease, and psychiatric disorders such as anxiety and depression, all of which contribute to reduced health-related quality of life (1, 3).

The etiology of PCOS is multifactorial, involving a complex interplay of endocrine, metabolic, immunological, and genetic factors; however, interactions among these factors remain poorly understood. No definitive cure exists for PCOS. For infertility, ovulation induction remains the primary treatment strategy (4). First-line agents such as clomiphene citrate or letrozole are widely prescribed, and gonadotropins are used when these treatments fail. However, gonadotropin therapy carries a risk of ovarian hyperstimulation syndrome (OHSS). In women with PCOS, gonadotropin administration frequently induces multifollicular growth (5), and subsequent hCG-triggered ovulation may increase vascular endothelial growth factor production in luteinized granulosa cells (GCs), enhancing vascular permeability and causing third-space fluid shift, thereby increasing the risk of OHSS (6, 7). Clinical studies have reported that the incidence of OHSS is markedly higher in patients with PCOS than in the general population (5). Beyond infertility management, lifestyle modification is the foundation of PCOS care, and hormone therapy is often required to regulate menstrual cycles or treat amenorrhea. In clinical practice, combined oral contraceptive pills are commonly prescribed to regulate menses and improve hyperandrogenic symptoms (3, 8–10). Additional treatment options include lifestyle changes, pharmacology, ovarian drilling, and *in vitro* fertilization, although the latter also carries OHSS risks (10).

Bone morphogenetic proteins (BMPs), which belong to the transforming growth factor-β (TGF-β) superfamily, play essential roles in female reproductive function, including folliculogenesis, ovarian physiology, and fertility (11). BMP4, in particular, regulates follicular growth and steroid hormone synthesis, and impaired BMP4 signaling has been implicated in PCOS pathogenesis (12). Therefore, BMP4 is considered a promising therapeutic target in PCOS.

Herbal medicine has recently gained attention as a complementary treatment for PCOS, with growing evidence supporting its roles in hormonal regulation and symptom improvement (9). Clinical (13) and basic research (14, 15) suggest that traditional Japanese medicines may ameliorate PCOS-related abnormalities.

Tokishakuyakusan (TSS) is a traditional Japanese herbal formulation widely used to treat gynecological disorders. In Eastern Asia, it is also known as Danggui Shaoyao San or Danggwijagyaksan. Clinical evidence supports its efficacy in primary dysmenorrhea (16, 17) and menopausal symptoms (18). TSS in combination with low-dose oral contraceptives has demonstrated benefit in patients with endometriosis and dysmenorrhea (19). Additionally, a database study reported that Japanese Kampo medicines, including TSS, are associated with increased live birth rates in women with recurrent pregnancy loss (20). Animal studies have also shown that TSS improves implantation failure (21), alleviates preeclampsia (22), reduces hyperalgesia and lesion formation in endometriosis (23), enhances oviductal contractility (24), and ameliorates cold-induced hypothermia by promoting early blood flow recovery (25).

Despite growing evidence supporting its therapeutic potential, the effects of TSS on PCOS and the underlying mechanisms remain unclear. Elucidating these mechanisms may reveal new therapeutic possibilities for PCOS.

Therefore, in this study, we investigated the efficacy of TSS on PCOS-like phenotypes, including irregular estrous cycles and ovarian morphological abnormalities, using a prenatal 5α-dihydrotestosterone (DHT) exposure rat model, which mimics the lean PCOS phenotype commonly observed in Asian women (26). Comprehensive gene expression analyses and subsequent validation experiments further identified BMP signaling as a key contributor to the observed effects.

## 2 Materials and methods

### 2.1 Drugs and reagents

DHT was purchased from Tokyo Chemical Industry (Tokyo, Japan), dissolved in ethanol (Kokusan Chemical Co., Ltd., Tokyo, Japan), diluted to 20% with sesame oil (Sigma-Aldrich, St. Louis, MO, USA), and adjusted to a final concentration of 15 mg/mL. The TSS powder extract (Lot No. 2220023020) was obtained from TSUMURA & CO. (Tokyo, Japan), a manufacturer of traditional Japanese medicines approved for clinical use by the Japanese Ministry of Health, Labour and Welfare. TSS was produced by extracting a mixture of six crude drugs that met the Japanese Pharmacopoeia quality standards (**Table 1**) with water at 95°C for 1 h, followed by filtration to remove insoluble material. The extract was concentrated under reduced pressure, spray-dried, and processed into powdered TSS extract. **Supplementary Figure 1** shows the components of TSS identified via three-dimensional high-pressure liquid chromatography.

**Table 1:** Formula of Tokishakuyakusan.

Crude drug	Origin	Weight (g)
Peony root	*Paeonia lactiflora* Pallas, *Paeoniaceae* (root)	4
*Atractylodes lancea* rhizome	*Atractylodes lanceae* De Candolle or *Atractylodes chinensis* Koidzumi, *Compositae* (rhizome)	4
Alisma tuber	*Alisma orientale* Juzepczuk*, Alismataceae* (tuber)	4
*Poria* sclerotium	*Wolfiporia cocos* Ryvarden et Gilbertson (*Poria cocos* Wolf), *Polyporaceae* (sclerotium)	4
*Cnidium* rhizome	*Cnidium officinale* Makino, *Umbelliferae* (rhizome)	3
Japanese *Angelica* root	*Angelica acutiloba* Kitagawa or *Angelica acutiloba* Kitagawa var. *sugiyamae* Hikino, *Umbelliferae* (root)	3

### 2.2 Animals

Seven pregnant Wistar rats were purchased from Jackson Laboratory Japan (Yokohama, Japan). Four rats were used for animal experiments and three for primary GC culture. Rats used in the animal experiments were 10 weeks of age and at gestational day 8 at purchase, whereas those used for GC culture were 9 weeks of age and at gestational day 13. All animals, including their offspring, were housed in appropriately sized cages in a controlled environment (20°C–25°C; 12-h light/dark cycle) with free access to tap water and either CE-2 diet (CLEA Japan, Tokyo, Japan) or MF diet (Oriental Yeast, Tokyo, Japan). All animal procedures were approved by the Animal Experiment Committee of Nagoya University Graduate School of Medicine (M240057-002) and by TSUMURA & CO. (Approval No. 23-002). Animal care and use complied with institutional guidelines and relevant regulations.

### 2.3 Experimental design

**Figure 1:**
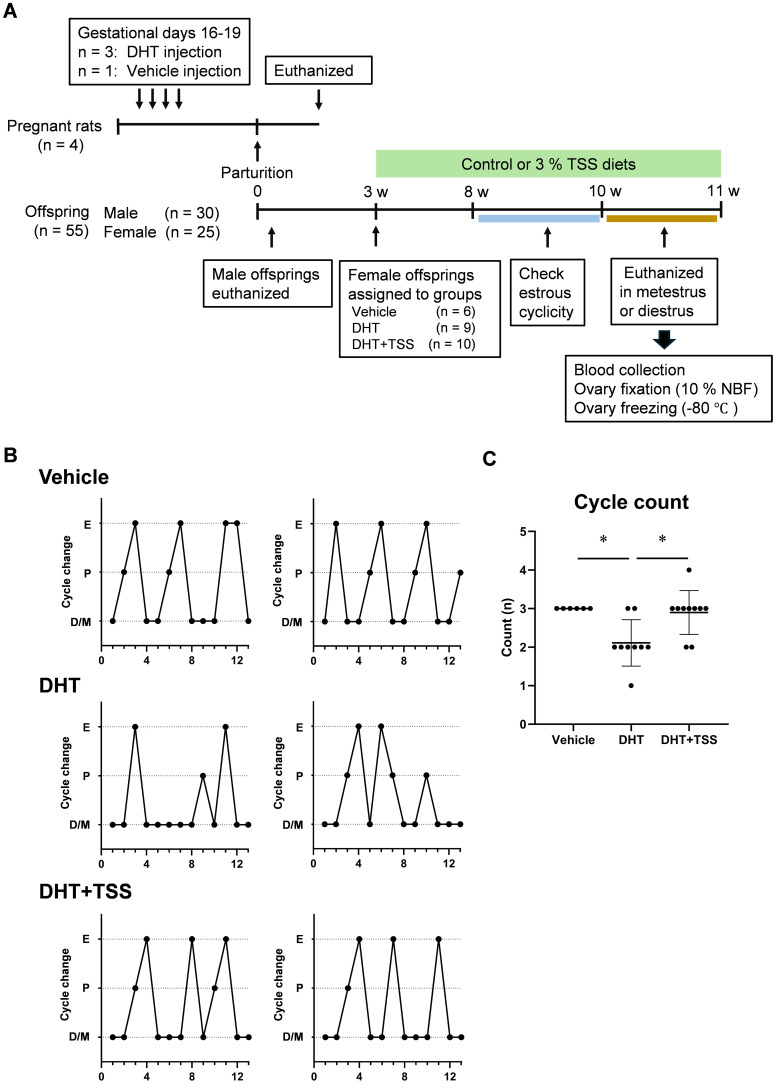
Experimental design and estrous cycle. **(A)** Timeline of the *in vivo* experimental design. A PCOS model was developed via prenatal DHT treatment in rats. Female offspring (3 weeks old) were fed a CE-2 diet (control diet) or 3% TSS-containing CE-2 diet. **(B)** Representative estrous cycles in each group. **(C)** Cycle count. *P < 0.05, Kruskal–Wallis test followed by Dunn’s test. D, diestrus; P, proestrus; E, estrus; M, metestrus; DHT, 5α-dihydrotestosterone; TSS, Tokishakuyakusan.

A schematic representation of the animal experiments is shown in **Figure 1A**. Of the four pregnant rats, three received DHT solution (3 mg/rat) and one received vehicle (40 μL 100% ethanol + 160 μL sesame oil) subcutaneously once daily from gestational day 16 for 4 consecutive days. Female offspring prenatally exposed to DHT were used as PCOS rats (14, 26). At 3 weeks of age, the female offspring of vehicle-treated mother rats were assigned to the vehicle group (*n* = 6), whereas the female offspring of DHT-treated mother rats were randomly assigned to the DHT (*n* = 9) and DHT+TSS (*n* = 10) groups. TSS was mixed with CE-2 diet at 3%, prepared by CLEA Japan. This concentration corresponded to approximately six times the human equivalent dose, based on previous reports (21). Rats were housed in groups of three to four per cage. The DHT+TSS group received the TSS-mixed diet, whereas the DHT and vehicle groups received a similarly prepared TSS-free CE-2 diet ad libitum. Based on food intake and weight gain, the estimated TSS dosage varied by approximately 5%.

Estrous cycles were assessed daily in offspring between 8 and 10 weeks of age via vaginal smears classified as proestrus, estrus, metestrus, or diestrus (27). At 10–11 weeks of age, female offspring were euthanized during diestrus or metestrus. Under isoflurane anesthesia, blood samples were collected by cardiac puncture, and serum was isolated by centrifugation (3,000×*g*, 10 min). Both ovaries were removed; one was fixed in 10% neutral-buffered formalin, and the other was stored at −80°C.

### 2.4 Serum hormone level measurements

Serum estradiol (E2), progesterone (P4), and testosterone (T) levels were measured using electrochemiluminescence immunoassays performed by SRL Inc. (Tokyo, Japan) following validated standard protocols. Serum follicle-stimulating hormone (FSH) and luteinizing hormone (LH) levels were determined using the LBIS Rat FSH ELISA Kit (AKRFS-010, FUJIFILM Wako Shibayagi, Gunma, Japan) and Rat LH ELISA Kit (S-type, AKRLH-010S, FUJIFILM Wako Shibayagi), respectively, according to the manufacturers’ instructions. All samples were assayed in duplicate. The limits of quantitation were 0.133 ng/mL for FSH and 0.153 ng/mL for LH. Standard assay ranges were 0.4–20 ng/mL (FSH) and 0.313–10 ng/mL (LH). Manufacturer-reported intra- and inter-assay coefficients of variation were <10% for FSH and <5% for LH. Standard curves were generated using supplied calibrators (four-parameter logistic model).

### 2.5 Analysis of ovarian morphology

Formalin-fixed ovaries were paraffin-embedded, sectioned at 6 µm, and stained with hematoxylin and eosin. Ovarian follicles were classified into six categories: primary, secondary, small antral, large antral, preovulatory, and atretic cyst-like follicles, according to established criteria (**Table 2**) (26, 28). Six representative sections per ovary, spaced at least 300 µm apart, were analyzed. Follicle counts were performed using a light microscope (Axio Imager A1; Carl Zeiss, Oberkochen, Germany). Entire ovaries were examined to avoid double-counting large follicles or corpora lutea, scanning multiple sections. Total follicle counts excluded corpora lutea, as previously described (26).

**Table 2:** Classification of ovarian follicles.

Follicle type	Definition
Primary	Oocyte with one layer of cuboidal granulosa cells (GCs)
Secondary	Oocyte with two to five layers of GCs
Small antral	Oocyte surrounded by more than five layers of GCs, with one or two small pockets of follicular fluid (or both)
Large antral	Follicle containing a single large antral cavity
Preovulatory	Follicle with a single large antrum and an oocyte surrounded by cumulus cells attached to the mural GC layer
Atretic cyst-like	Large fluid-filled cyst with an attenuated GC layer and a dispersed theca cell layer

### 2.6 Primary culture of ovarian GCs

GCs were obtained as previously described with minor modifications (29). Three pregnant rats received vehicle (*n* = 1) or DHT (*n* = 2). Female offspring (3 weeks old, *n* = 4 per experiment) were injected subcutaneously with pregnant mare serum gonadotropin (10 IU; Aska Animal Health, Tokyo, Japan). After 48 h, the ovaries were collected under deep isoflurane anesthesia. GCs were isolated by puncturing follicles with a 27-gauge needle under a stereomicroscope and filtering the aspirate through 100- and 40-μm strainers (Corning, NY, USA).

GCs (3 × 10^5^ cells/well) were seeded into 6-well plates (Corning Primaria, Corning) and cultured at 37°C with 5% CO_2_ in phenol red-free Dulbecco’s modified Eagle’s medium and Ham’s F-12 medium (DMEM/F12 medium; Thermo Fisher Scientific, Waltham, MA, USA) supplemented with 10% fetal bovine serum (JRH Biosciences, Lenexa, KS, USA) and penicillin–streptomycin solution (100 IU/mL and 100 μg/mL; Thermo Fisher Scientific).

After 48 h of incubation, the medium was replaced with serum-free DMEM/F12 medium supplemented with 1% non-essential amino acid solution (FUJIFILM Wako Chemicals, Osaka, Japan) and 0.1% bovine serum albumin (Sigma-Aldrich, St. Louis, MO, USA). To prevent luteinization, the medium contained final concentrations of 10 ng/mL of insulin (Sigma-Aldrich), 2.5 μg/mL of transferrin (Nacalai Tesque, Kyoto, Japan), and 4 ng/mL of sodium selenite (Wako). To examine the effects of TSS, GCs were treated with TSS (125, 250, and 500 μg/mL) and/or FSH (3 ng/mL; R&D Systems, Minneapolis, MN, USA) and cultured for 24 h in a medium supplemented with 100 nmol/L of androstenedione (Tokyo Chemical Industry).

### 2.7 Gene expression

#### 2.7.1 Quantitative real-time polymerase chain reaction

Ovarian tissues stored at −80°C were homogenized in QIAzol Lysis Reagent (Qiagen, Venlo, Netherlands). Cultured GCs were washed with phosphate-buffered saline and lysed in QIAzol (Qiagen). RNA was extracted from tissue homogenates and cell lysates using the RNeasy Universal Tissue Kit (Qiagen). Complementary DNA was synthesized using the TaqMan High-Capacity Reverse Transcription Kit (Thermo Fisher Scientific).

Gene expression profiling of ovarian tissues was performed using customized 96-well TaqMan Array plates (Thermo Fisher Scientific) comprising 48 target genes, including the endogenous control 18S ribosomal RNA (*18s*) (**Supplementary Table**). Quantitative real-time polymerase chain reaction (qPCR) was used to analyze target gene expression in ovarian tissues and cultured GCs. Thermal cycling conditions were as follows: initial hold at 95°C for 20 s, followed by 40 cycles of denaturation at 95°C for 1 s and annealing/extension at 60°C for 20 s. mRNA levels were quantified using the QuantStudio 7 Flex Real-Time PCR System (Thermo Fisher Scientific) with TaqMan Fast Advanced Master Mix (Thermo Fisher Scientific) and normalized to *18s* or *glyceraldehyde-3-phosphate dehydrogenase* (*Gapdh*). The following TaqMan primer–probe sets (Thermo Fisher Scientific) were used: *Bmp4*, Rn00432087_m1; *Bmp5*, Rn01447676_m1; *inhibin-βa* (*Inhba*), Rn01538592_m1; *steroidogenic acute regulatory protein* (*Star*), Rn00580695_m1; *cytochrome P450 11A1* (*Cyp11a1*), Rn00568733_m1; *3β hydroxysteroid dehydrogenase* (*Hsd3b*), Rn01789220_m1; *18s*, Hs99999901_s1; and *Gapdh*, Rn01775763_g1.

#### 2.7.2 Western blotting

Ovarian tissues stored at −80°C were homogenized in RIPA buffer (FUJIFILM Wako Chemicals) supplemented with protease inhibitor cocktail (Sigma-Aldrich) and Phosphatase Inhibitor Cocktail Tablets (PhosSTOP; Sigma-Aldrich). Lysates were centrifuged twice at 10,000×*g* for 10 min at 4°C. Supernatants (20 μg protein/lane) were separated via sodium dodecyl sulfate-polyacrylamide gel electrophoresis (200 V, 40 min) and transferred to polyvinylidene difluoride membranes (30 V, 60 min). Membranes were blocked for 1 h at room temperature with SuperBlock Blocking Buffer in TBS (37535; Thermo Fisher Scientific) and incubated overnight at 4°C with primary antibodies against BMP4 (MAB1049; 1:1,000, Merck Millipore, Burlington, MA, USA) or inhibin βA (17524-1-AP; 1:1,000, Proteintech Group, Inc., Rosemont, IL, USA). After washing, membranes were incubated for 1 h at room temperature with HRP-conjugated secondary antibodies (ECL Anti-Mouse IgG, NA9310V; 1:20,000, or ECL Anti-Rabbit IgG, NA934VS; 1:100,000; Cytiva, Marlborough, MA, USA). For β-actin detection, membranes were stripped using Restore Stripping Buffer (21059; Thermo Fisher Scientific), re-probed with anti-β-actin antibody (sc-47778; 1:20,000, Santa Cruz Biotechnology, Dallas, TX, USA), and incubated with ECL Anti-Mouse IgG (NA9310V; 1:20,000; Cytiva). Protein signals were visualized using ECL Prime (Cytiva) and imaged on a FUSION FX system (Vilber Bio Imaging, Collegien, France).

### 2.8 Progesterone measurements in culture medium

Progesterone concentrations in culture supernatants from GCs treated with TSS (500 μg/mL) and/or FSH (3 ng/mL) for 24 h were measured using a progesterone ELISA kit (ADI-900-011; Enzo Life Sciences, Farmingdale, NY, USA).

### 2.9 Statistical analysis

Data are presented as line or bar graphs and expressed as mean, standard deviation, and individual data points. Normality was assessed using the Shapiro–Wilk test in the R software (version 4.3.1; R Foundation for Statistical Computing, Vienna, Austria, https://www.R-project.org/). For parametric data, statistical significance was tested using one- or two-way analysis of variance (ANOVA) followed by Dunnett’s or Tukey’s *post hoc* multiple comparison test. For non-parametric data, the Kruskal–Wallis test followed by Dunn’s test was used. Simple linear regression was performed for correlation analyses. Statistical significance was defined as *P <*0.05 (Dunnett’s, Tukey’s, and Dunn’s tests) or *P <*0.0167 (Student’s *t*-test with Bonferroni correction). Analyses were performed using GraphPad Prism version 8.4.3 (GraphPad Software, San Diego, CA, USA). Gene expression profiling (volcano plot analysis) was conducted using the tidyverse package in R, and differences between two groups were assessed using Welch’s *t*-test.

## 3 Results

### 3.1 TSS alleviates abnormal ovarian morphology in a PCOS rat model

We evaluated the effects of TSS on PCOS-like phenotypes in prenatally DHT-treated rats (**Figure 1A**). Representative estrous cycles assessed by vaginal smears are shown in **Figure 1B**. Rats in the vehicle group exhibited regular estrous cycles, whereas those in the DHT group displayed irregular cycles characterized by persistent diestrus. In contrast, the estrous cycle in the DHT+TSS group was improved relative to the DHT group (**Figure 1C**).

**Figure 2:**
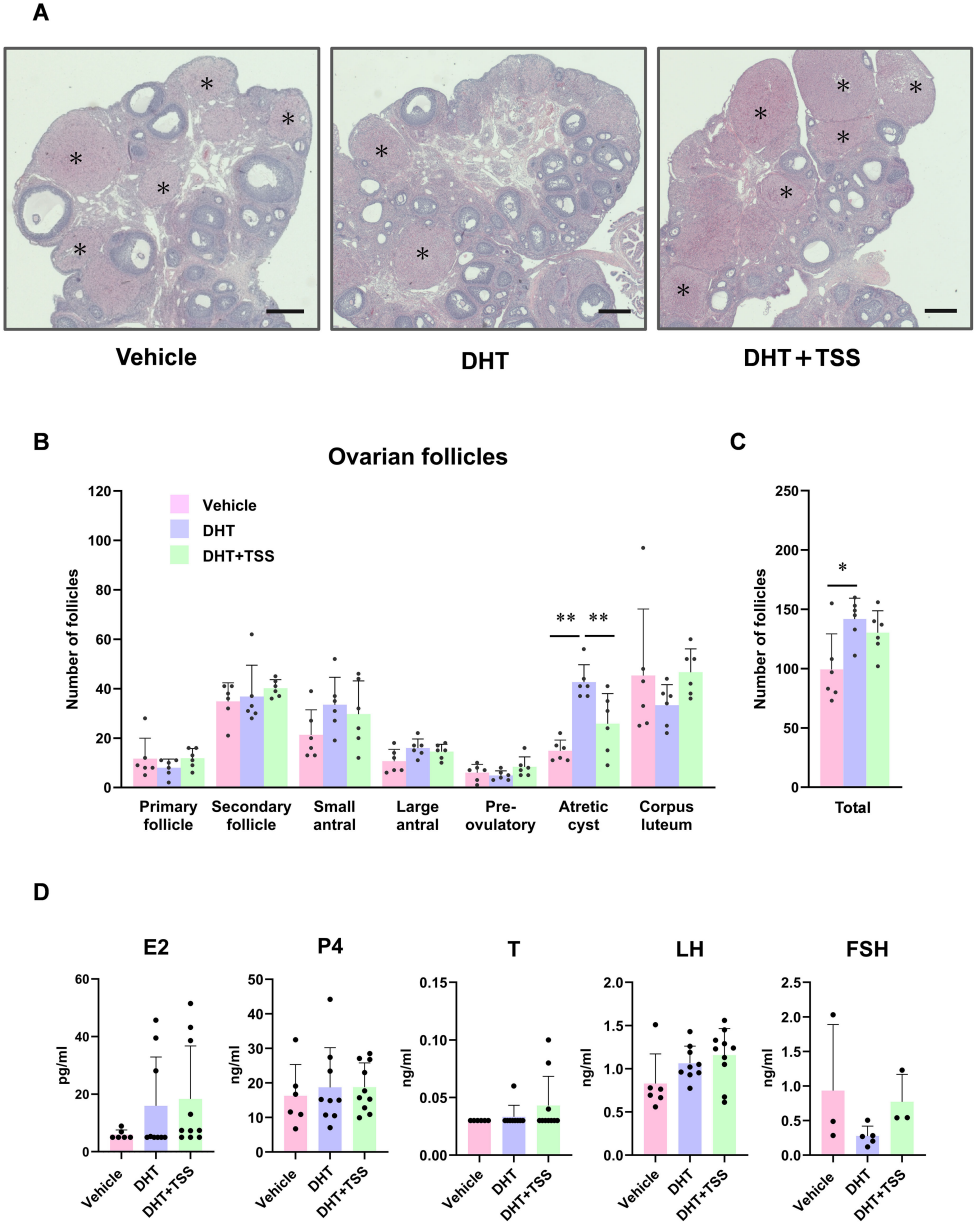
Effect of Tokishakuyakusan (TSS) on ovarian morphology in a PCOS rat model. **(A)** Representative ovarian morphology (hematoxylin and eosin; scale bar: 1 mm; original magnification ×25). The asterisk indicates a corpus luteum. **(B)** Number of ovarian follicles at each follicle stage, atretic cyst-like follicles, and corpus luteum. **(C)** Total number of ovarian follicles and atretic cyst-like follicles. Data are expressed as mean ± standard deviation with individual data points (*n* = 6 per group). **(D)** Serum concentrations of steroid hormones and gonadotropins. Data are presented as mean ± standard deviation with individual points (vehicle: *n* = 6; DHT: *n* = 9; DHT+TSS: *n* = 10). *P < 0.05, **P < 0.01, one-way analysis of variance (ANOVA) followed by Tukey’s *post hoc* test; *P* < 0.0167 by Bonferroni correction. DHT, 5α-dihydrotestosterone.

Representative ovarian morphologies from the vehicle, DHT, and DHT+TSS groups are presented in **Figure 2A**. Compared with the vehicle and DHT+TSS groups, a trend toward decreased corpora lutea was observed in the DHT group. Therefore, we quantified ovarian follicles at each developmental stage, atretic cyst-like follicles, and corpora lutea. No significant differences in corpora lutea counts were detected among the groups (**Figure 2B**). The number of atretic cyst-like follicles was significantly higher in the DHT group than in the vehicle group (*P* < 0.0001), and this increase was alleviated in the DHT+TSS group (*P* < 0.01) (**Figure 2B**). No significant differences in other follicle types were observed between the DHT and DHT+TSS groups. The total follicle count was significantly higher in the DHT group than in the vehicle group (*P* = 0.0144) (**Figure 2C**).

We next measured gonadotropins and steroid hormone concentrations during diestrus and metestrus. Although serum LH levels in the DHT group tended to be higher than those in the vehicle group, no statistically significant differences were detected in serum E2, P4, T, LH, or FSH among the groups (**Figure 2D**). Some samples fell below the quantification limit for FSH; nevertheless, detectable FSH levels exhibited a negative correlation with ovarian *Inhba* expression (*P* = 0.0682) and with the number of atretic cyst-like follicles (*P* = 0.0071) (**Supplementary Figure 2**).

### 3.2 Ovarian *Bmp4* expression correlates with ovarian morphology in a PCOS rat model

**Figure 3:**
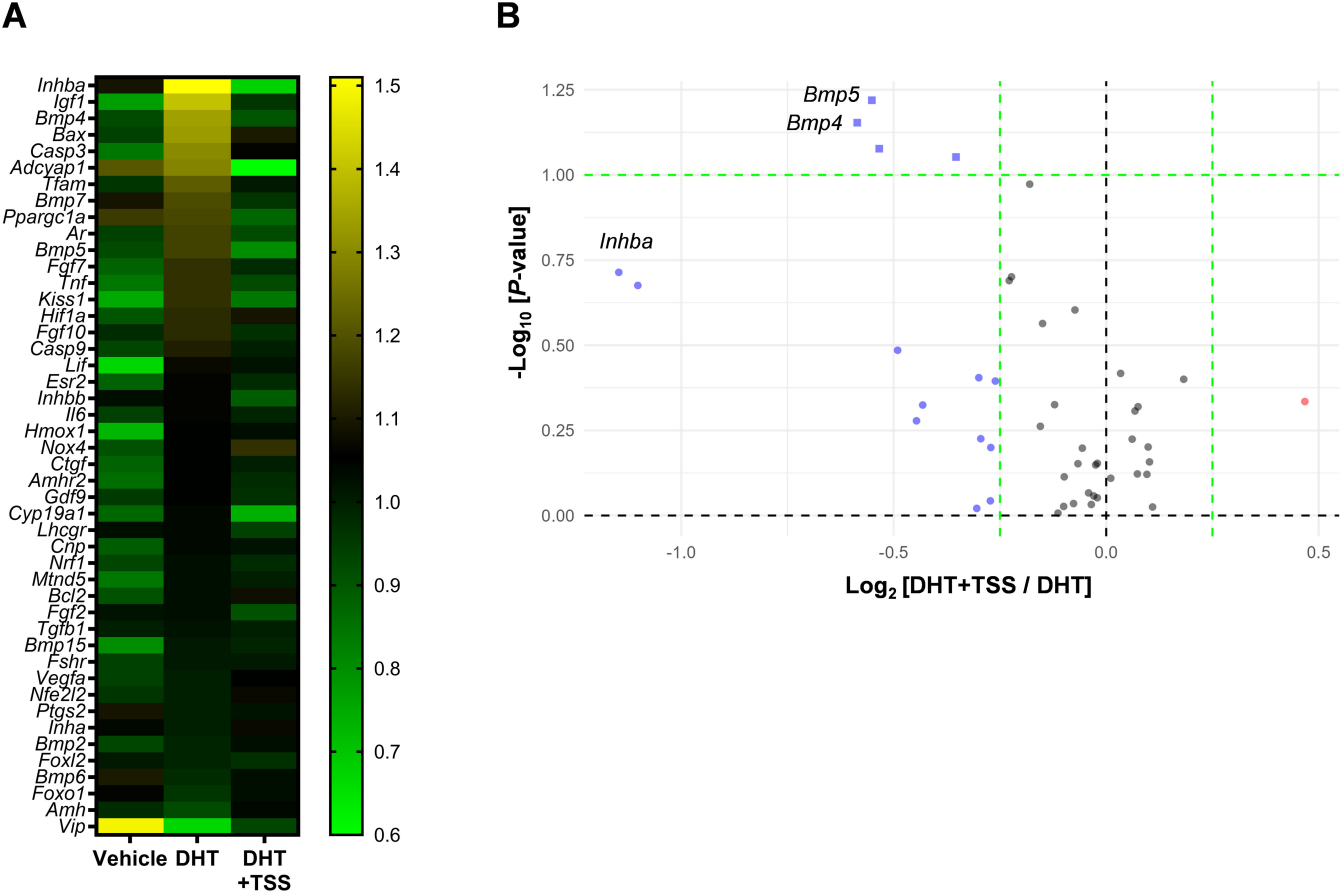
Gene expression profile in ovarian tissues. **(A)** Heatmap showing the median fold change of each gene in ovarian tissues of the vehicle (*n* = 6), DHT (*n* = 9), and DHT+TSS (*n* = 10) groups. Yellow indicates upregulation and green indicates downregulation relative to the median for each gene. **(B)** Volcano plot of differential gene expression between the DHT+TSS (*n* = 10) and DHT (*n* = 9) groups. Color and shape indicate upregulation (red), downregulation (blue), *P* < 0.1 (square), and *P* ≥ 0.1 (circle). DHT, 5α-dihydrotestosterone; TSS, Tokishakuyakusan.

**Figure 4:**
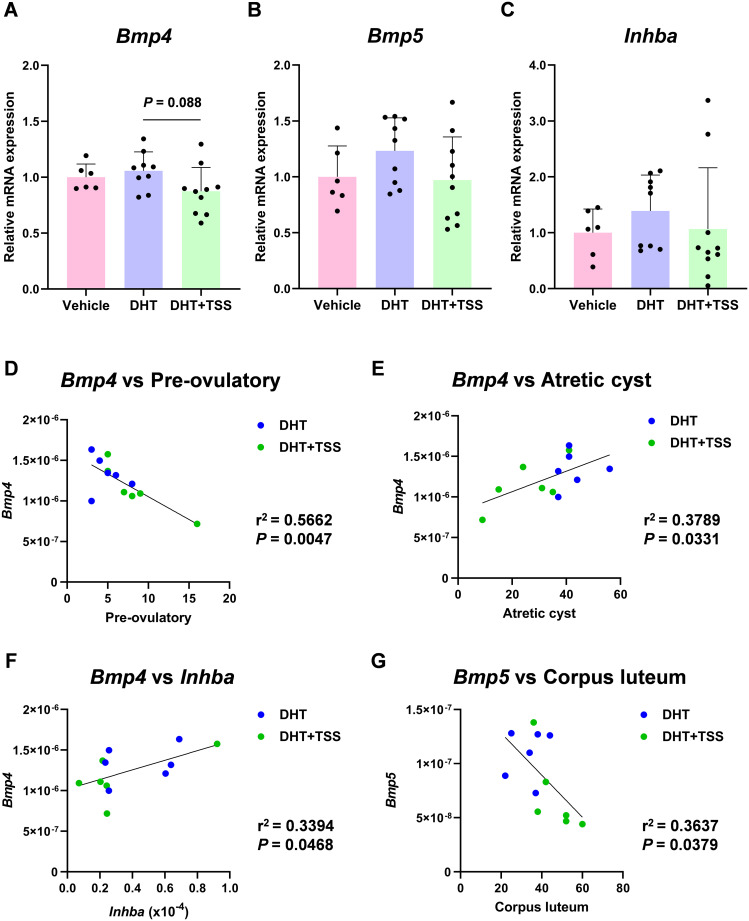
*Bmp4*, *Bmp5*, and *Inhba* expression in the ovaries. **(A–C)** Relative mRNA expression quantified by real-time PCR. Expression levels are relative to 18S ribosomal RNA. Data are shown as mean ± standard deviation with individual data points. *P*-values were obtained by one-way analysis of variance (ANOVA) with Tukey’s *post hoc* test (vehicle: *n* = 6; DHT: *n* = 9; DHT+TSS: *n* = 10). **(D–G)** Correlation analysis between gene expression and ovarian follicle counts in the DHT and DHT+TSS groups (*n* = 6 per group). The correlation coefficient (*r*^2^) and *P*-value were calculated. DHT, 5α-dihydrotestosterone; TSS, Tokishakuyakusan; Bmp, bone morphogenetic protein; Inhba, inhibin-βa; PCR, polymerase chain reaction.

**Figure 5:**
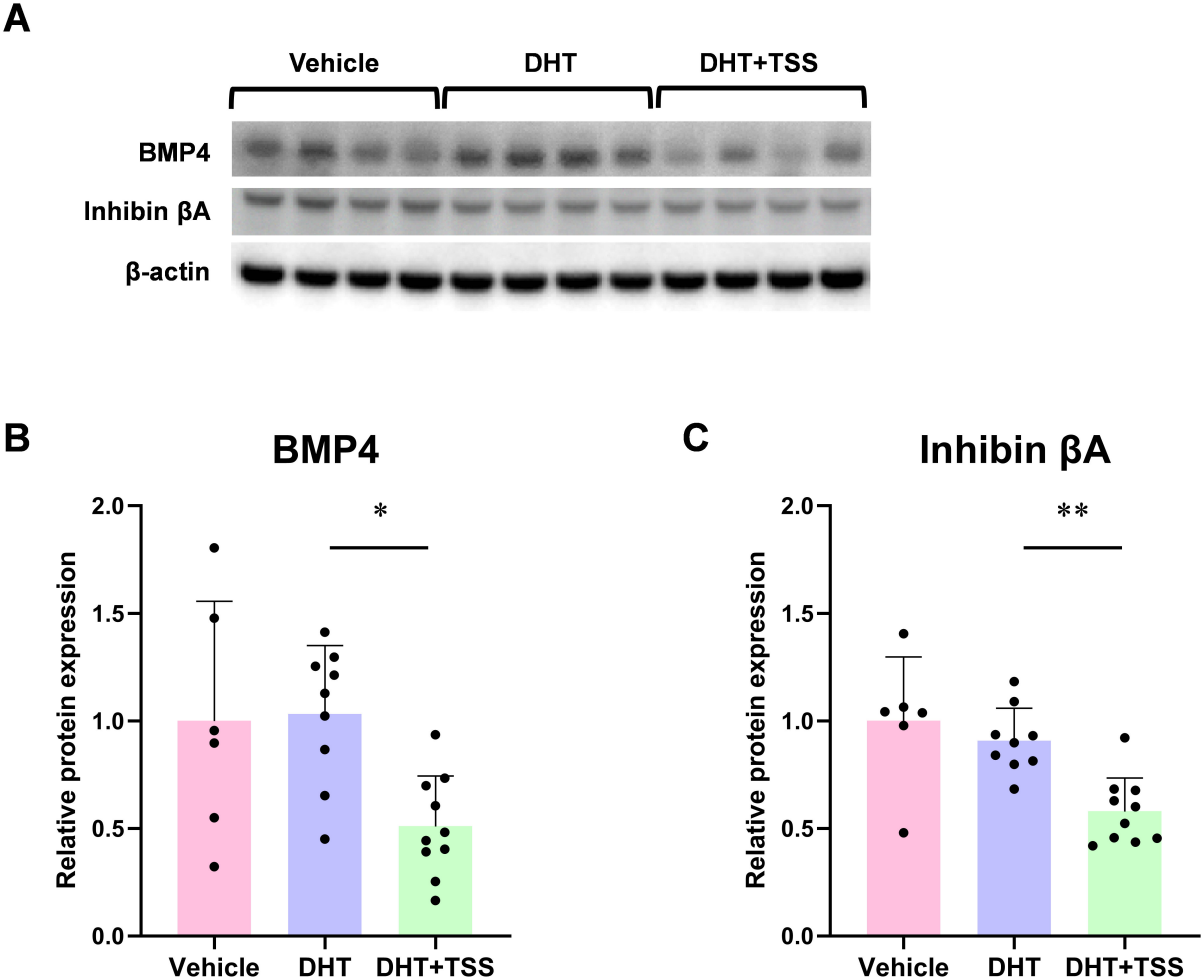
BMP4 and inhibin βA protein expression in the ovaries. **(A)** Representative Western blot images. **(B, C)** Quantification of BMP4 and inhibin βA protein levels relative to β-actin. Data are shown as mean ± standard deviation with individual data points (vehicle: *n* = 6; DHT: *n* = 9; DHT+TSS: *n* = 10). *P < 0.05, **P < 0.01, one-way analysis of variance (ANOVA) followed by Tukey’s *post hoc* test; *P* < 0.0167 by Bonferroni correction. BMP, bone morphogenetic protein; DHT, 5α-dihydrotestosterone; TSS, Tokishakuyakusan.

To investigate whether TSS affects reproductive regulators involved in follicle development and ovarian function, we performed gene expression profiling of ovarian tissues using TaqMan Array plates, targeting 46 genes. Differentially expressed genes were identified using heatmap (**Figure 3A**) and volcano plot analyses (**Figure 3B**). The DHT+TSS group showed decreased trends in *Bmp4*, *Bmp5*, and *Inhba* relative to the DHT group, and these trends were confirmed by additional qPCR analyses (**Figures 4A–C**). Correlation analyses in PCOS rats showed that *Bmp4* expression was negatively correlated with the number of preovulatory follicles and positively correlated with atretic cyst-like follicles and *Inhba* expression (**Figures 4D–F**). A significant correlation was also observed between *Bmp5* and the number of corpora lutea (**Figure 4G**). These results suggest that TSS-induced follicular development is associated with reduced *Bmp4* and *Bmp5* expression. Western blot analysis demonstrated significantly decreased protein levels of BMP4 and inhibin βA in ovarian tissues from the DHT+TSS group compared with the DHT group (**Figure 5**; **Supplementary Figure 3**).

### 3.3 TSS regulates *Bmp4* expression and steroidogenesis in primary cultured GCs

**Figure 6:**
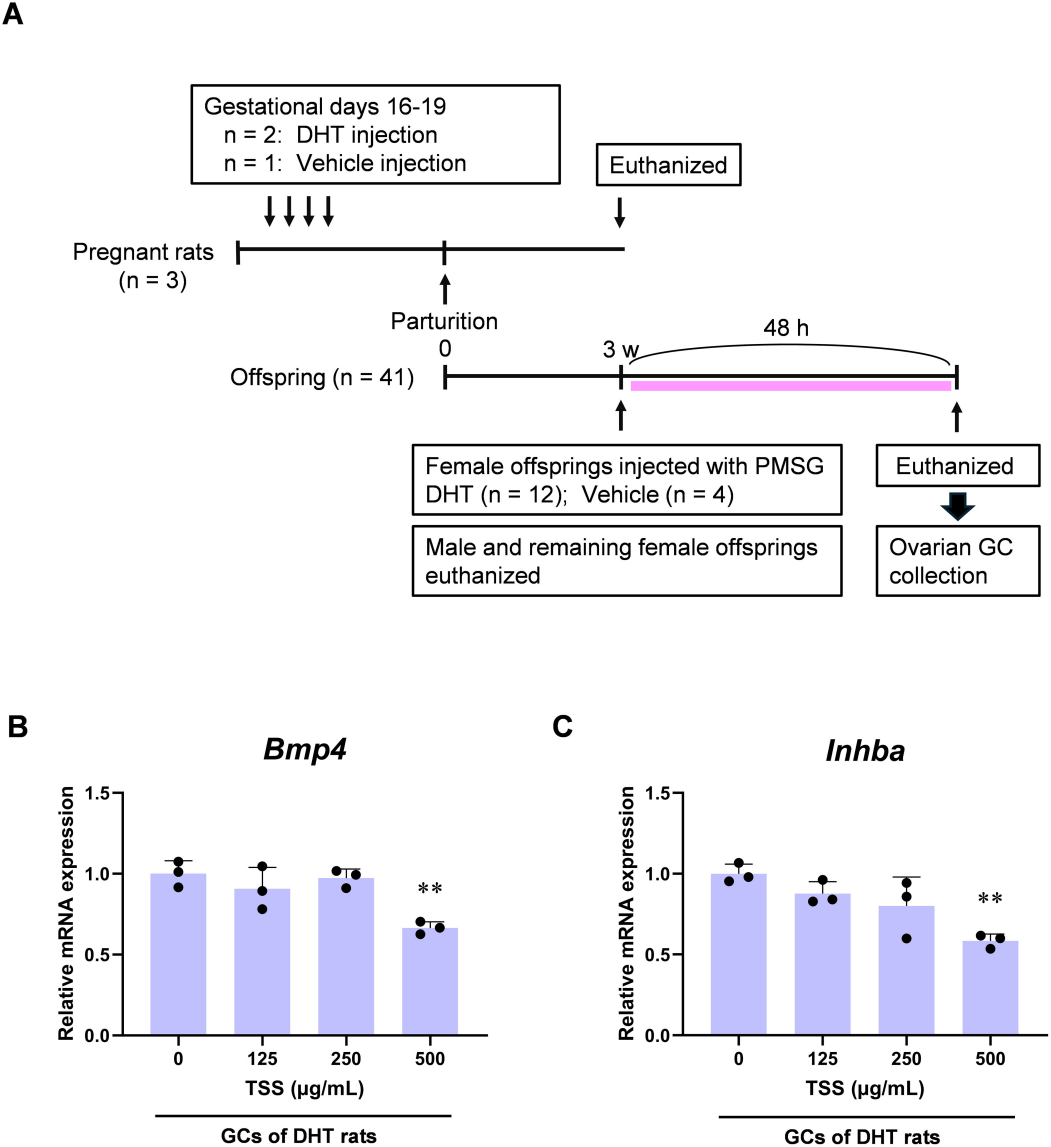
Effect of Tokishakuyakusan (TSS) on target gene expressions in granulosa cells (GCs) derived from prenatally 5α-dihydrotestosterone (DHT)-treated rats. **(A)** Timeline of ovarian GC collection. **(B, C)** Relative mRNA expression of *Bmp4* and *Inhba* in primary cultured GCs after 24 h of TSS treatment (125–500 μg/mL). Expression levels are relative to *Gapdh*. Data are shown as mean ± standard deviation with individual data points (*n* = 3 per group). **P < 0.01, *P*-values for the untreated control were obtained using one-way analysis of variance (ANOVA) followed by Dunnett’s *post hoc* test. PMSG, pregnant mare serum gonadotropin; Bmp, bone morphogenetic protein; Inhba, inhibin-βa; Gapdh, glyceraldehyde-3-phosphate dehydrogenase.

To clarify how TSS influences the target genes *Bmp4*, *Bmp5*, and *Inhba*, we examined their mRNA expression in primary cultured ovarian GCs obtained from the offspring of DHT-treated rats (**Figure 6A**). TSS treatment (500 µg/mL, 24 h) significantly decreased *Bmp4* expression in cultured GCs (**Figure 6B**). *Bmp5* expression in GCs was extremely low, preventing evaluation of group differences (data not shown). *Inhba* expression in GCs derived from DHT rats was also reduced following TSS treatment (**Figure 6C**).

**Figure 7:**
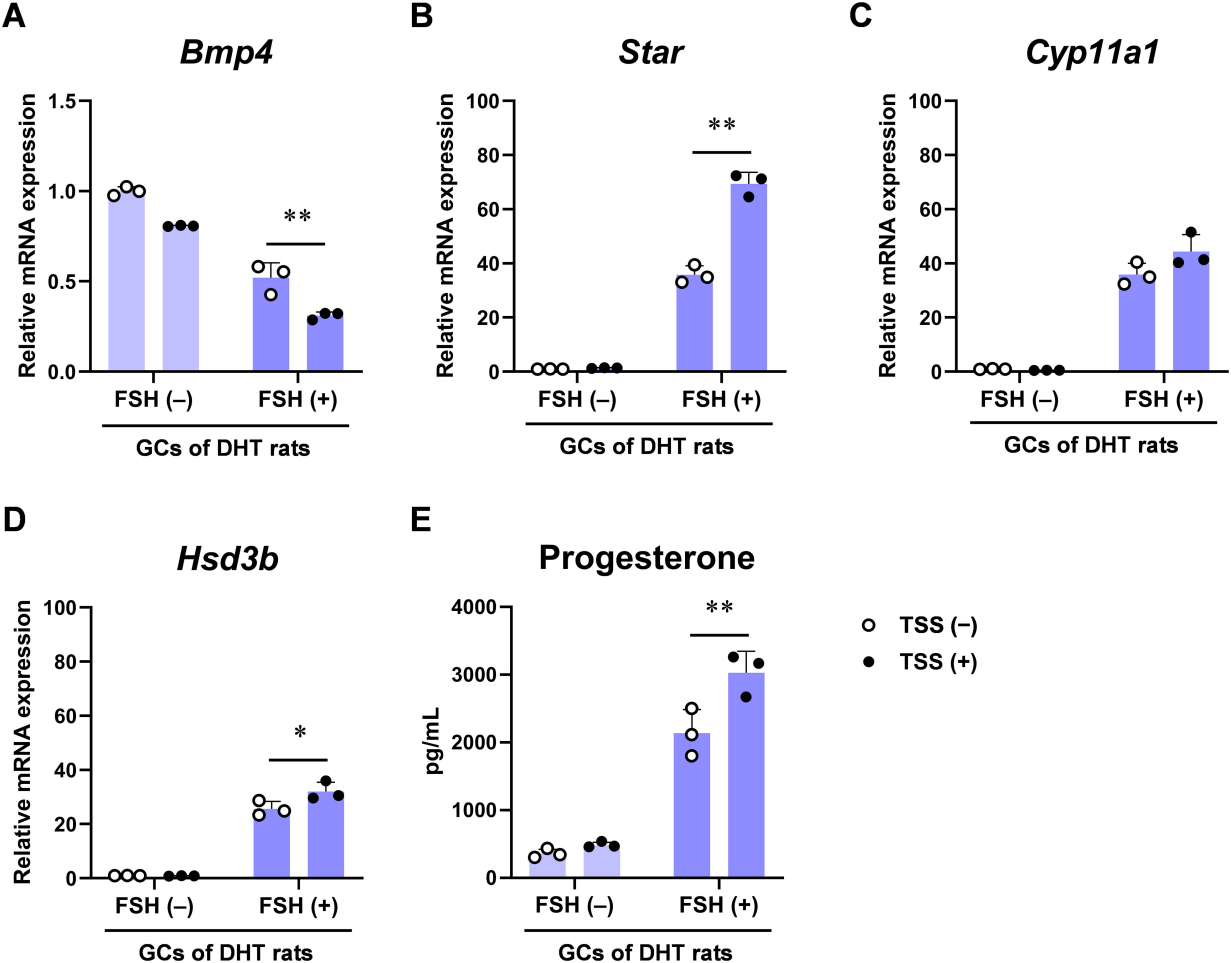
Effect of Tokishakuyakusan (TSS) on progesterone synthesis in granulosa cells (GCs) derived from prenatally 5α-dihydrotestosterone (DHT)-treated rats. **(A–D)** Relative mRNA expression of *Bmp4***(A)**, *Star***(B)**, *Cyp11a1***(C)**, and *Hsd3b***(D)** in primary cultured GCs after 24 h of TSS (500 μg/mL) treatment with or without FSH (3 ng/mL). Expression levels are relative to Gapdh. **(E)** Progesterone concentration in GC culture medium after 24 h of treatment. Data are shown as mean ± standard deviation with individual data points (*n* = 3 per group). *P < 0.05, **P < 0.01, two-way analysis of variance (ANOVA) followed by Tukey’s *post hoc* test. FSH, follicle-stimulating hormone; Star, steroidogenic acute regulatory protein; Cyp11a1, cytochrome P450 11A1; Hsd3b, 3β hydroxysteroid dehydrogenase; Gapdh, glyceraldehyde-3-phosphate dehydrogenase.

We next examined the effects of TSS on steroidogenesis. TSS (500 µg/mL) decreased *Bmp4* mRNA levels in GCs from DHT rats even in the presence of FSH (3 ng/mL) (**Figure 7A**). TSS significantly enhanced FSH-induced expression of *Star* and *Hsd3b*, but not *Cyp11a1* (**Figures 7B–D**). The effects of TSS on *Bmp4* and *Star* expression were dose-dependent (**Supplementary Figure 4**). TSS increased progesterone levels in the culture medium of GCs derived from DHT rats (**Figure 7E**), indicating enhanced FSH-induced progesterone production. TSS did not significantly alter gene expression in GCs derived from vehicle-treated offspring, although progesterone production slightly increased (**Supplementary Figure 5**).

## 4 Discussion

In this study, TSS administration improved PCOS-related phenotypes in a prenatally androgenized rat model that closely resembles the lean PCOS phenotype commonly observed in Asian women. Several methods have been used to generate rodent PCOS models, including the administration or implantation of androgens (testosterone, DHT, DHEA), estrogens, antiprogestins, or aromatase inhibitors (30). Among these, DHT is not aromatized to E2, allowing induction of PCOS-like phenotypes without confounding estrogenic effects. Prenatal androgen exposure is known to induce multiple PCOS-like characteristics in female offspring, including increased preantral and antral follicles, reduced preovulatory follicles and corpora lutea, and disrupted estrous cycles (30). Our previous study (26) showed that prenatally DHT-treated rats exhibit ovarian histopathological changes such as increased atretic cyst-like follicles, irregular estrous cycles, and elevated serum LH levels. Consistent with these findings, the present study demonstrated irregular estrous cycles and an increased number of follicles, particularly atretic cyst-like follicles, in prenatally DHT-treated rats. Importantly, TSS reduced the number of atretic cyst-like follicles and alleviated the irregular estrous cycles, suggesting a potential therapeutic effect of TSS in PCOS.

Relatively elevated LH levels and low FSH levels have been reported in patients with PCOS (31). In our study, serum gonadotropins and sex steroid hormone levels during diestrus and metestrus were not significantly different among the groups. Although LH tended to be higher in the DHT group than in the vehicle group, no significant difference was observed between the DHT and DHT+TSS groups, suggesting that TSS had minimal impact on LH secretion under these conditions. Because many serum FSH values were below the limit of quantification, definitive conclusions cannot be drawn; however, the LH/FSH ratio was lower in the DHT+TSS group. Overall, our endocrine data do not provide strong evidence of functional hormonal improvement, and further studies are needed to clarify the effects of TSS on gonadotropin regulation.

PCOS pathogenesis is strongly associated with alterations in the follicular environment that impair oocyte development (32). Numerous studies have shown that PCOS affects follicular development (33–35) and is associated with oxidative stress (36), inflammation (37, 38), mitochondrial dysfunction (39, 40), and other factors important for follicular and oocyte quality. To explore these aspects, we assessed ovarian gene expression profiles using TaqMan Array plates. Heatmap analysis revealed increased expression of *Inhba*, *insulin-like growth factor 1* (*Igf1*), and *Bmp4* in the DHT group. Volcano plot and qPCR analyses showed that *Bmp4*, *Bmp5*, *and Inhba* expression levels were lower in the DHT+TSS group, suggesting that TSS modulates genes involved in follicular development.

Western blot analysis further confirmed reduced BMP4 and inhibin βA protein expression in the DHT+TSS group. BMPs belong to the TGF-β superfamily, and dysregulated BMP4 expression is known to affect follicular development, PCOS pathogenesis, and even ovarian cancer (12). Elevated BMP4 levels have been reported particularly under hyperandrogenic conditions (41). In our PCOS model, *Bmp4* expression significantly correlated with multiple follicular parameters and with *Inhba* expression. These results support the possibility that TSS improves PCOS pathology partly through its effects on *Bmp4*.

Thus, we further investigated TSS effects using primary cultured GCs isolated from the ovaries of prenatally DHT-treated rats and found that *Bmp4* and *Inhba* levels were reduced by TSS treatment in the absence of FSH (**Figure 6**). This finding was consistent with the results from the ovaries of PCOS model rats. BMP4 is expressed in granulosa lutein and theca lutein cells (42). BMP4 also upregulates the production of inhibin βA subunits and increases the production of its constituent inhibin A and activin A in human luteinizing and bovine GCs (43). These findings suggest that the TSS-induced decrease in inhibin βA may be regulated by BMP4.

The activin–follistatin–inhibin system is important for female reproduction, and aberrations in this system can induce fertility problems, including PCOS. Locally, in the ovary, activin A stimulates the differentiation of primordial follicles into antral follicles while inhibiting steroid (P4, E2) formation in GCs (44). Inhibin A decreases FSH secretion from the pituitary via the blood circulation, whereas FSH promotes an increase in inhibin A. Thus, inhibin A and FSH mutually regulate secretion (45). The high inhibin secretory capacity in the ovaries of patients with PCOS is thought to be a cause of the differences in basal LH and FSH levels (46). These findings suggest that TSS may improve PCOS pathogenesis by reducing inhibin A production.

In human granulosa-lutein cells, BMP4 downregulates StAR expression through ALK3 and SMAD1/5/8-SMAD4 signaling pathways and inhibits gonadotropin-induced progesterone production (47). Notably, in GCs from prenatally DHT-treated rats, TSS treatment promoted the expression of progesterone synthases (*Star* and *Hsd3b*) and secretion of progesterone in the presence of FSH. Usuki S (48). has reported TSS effects on progesterone secretion, which may be at least partly related to a decrease in BMP4 expression. Accordingly, we support the hypothesis that TSS decreased BMP4 expression in the ovaries of PCOS model rats, which may have contributed to stimulating FSH-induced follicle development via regulating inhibin βA.

In clinical practice, inducing ovulation in patients with PCOS can be challenging. In some cases, follicular development cannot be achieved with clomiphene, letrozole, or ovarian stimulation by gonadotropins. In addition, patients with PCOS are at high risk of excessive follicle development, resulting in OHSS. Our results suggest that the use of TSS in combination with commonly used medications may improve effective follicle development and may be useful in improving pregnancy rates beyond general infertility treatment.

The present study has several strengths. First, by using an established prenatally DHT-treated model, we successfully reproduced an ovarian environment that reflects the pathophysiology of PCOS, including the lean PCOS phenotype that is frequently observed in Asian women, allowing a detailed evaluation of the effects of TSS under disease-relevant conditions. Using this model, we demonstrated that TSS administration reduced the number of atretic cyst-like follicles and improved estrous cycles, indicating a beneficial impact on follicular developmental abnormalities in PCOS. In addition, comprehensive gene expression analyses identified *Bmp4* and *Inhba* as novel target genes, and further examination in *in vivo* and *in vitro* systems clarified the effects of TSS on gene expression and steroidogenesis. Importantly, we also observed reductions in BMP4 and inhibin βA protein levels in ovarian tissues of the DHT+TSS group, supporting the notion that these molecules may be regulated by TSS under physiological conditions. Together, these findings suggest that TSS may exert favorable effects on PCOS pathophysiology and highlight its potential as a therapeutic option that could complement existing ovulation-induction treatments in clinical practice.

However, this study also has some limitations. It was limited to histopathological evaluation and estrous cycles, and functional reproductive data, such as ovulation rates or fertility outcomes, were not assessed. PCOS pathogenesis is associated with many factors, including the hypothalamic-pituitary-gonadal axis, insulin resistance, and immune abnormalities; however, the TSS effect on these factors is unknown. Moreover, although *Bmp4* expression was downregulated following TSS treatment, the identified relationship between BMP4 and the improvement of PCOS remains correlative rather than definitively causal. In the present study, we could not clarify whether the pharmacological effects were specific to PCOS. Further studies are required to elucidate the underlying mechanism. Future studies should consider multiple perspectives to validate our results.

In conclusion, in prenatally DHT-treated rats, TSS ameliorated PCOS-like histopathology characterized by increased atretic cyst-like follicles. Genetic and/or protein analyses of ovarian tissues and primary cultured GCs identified BMP4 and inhibin βA as target genes of TSS. These findings suggest that the regulation of BMP4 and inhibin βA expression by TSS may contribute to the improvement of PCOS-like histopathological changes.

## Data Availability

The original contributions presented in the study are included in the article/**Supplementary Material**. Further inquiries can be directed to the corresponding author.
