# Supplementary material for: Amelioration of polycystic ovarian morphology by Tokishakuyakusan in a PCOS rat model: association with bone morphogenetic protein 4
Source: Front Endocrinol (Lausanne). 2026 Jan 9;16:1649124. doi: 10.3389/fendo.2025.1649124 (PMC12827122; doi:10.3389/fendo.2025.1649124)
Supplement: Supplementary file 1 [file DataSheet1.docx]

Supplementary Material

## Supplementary Figures


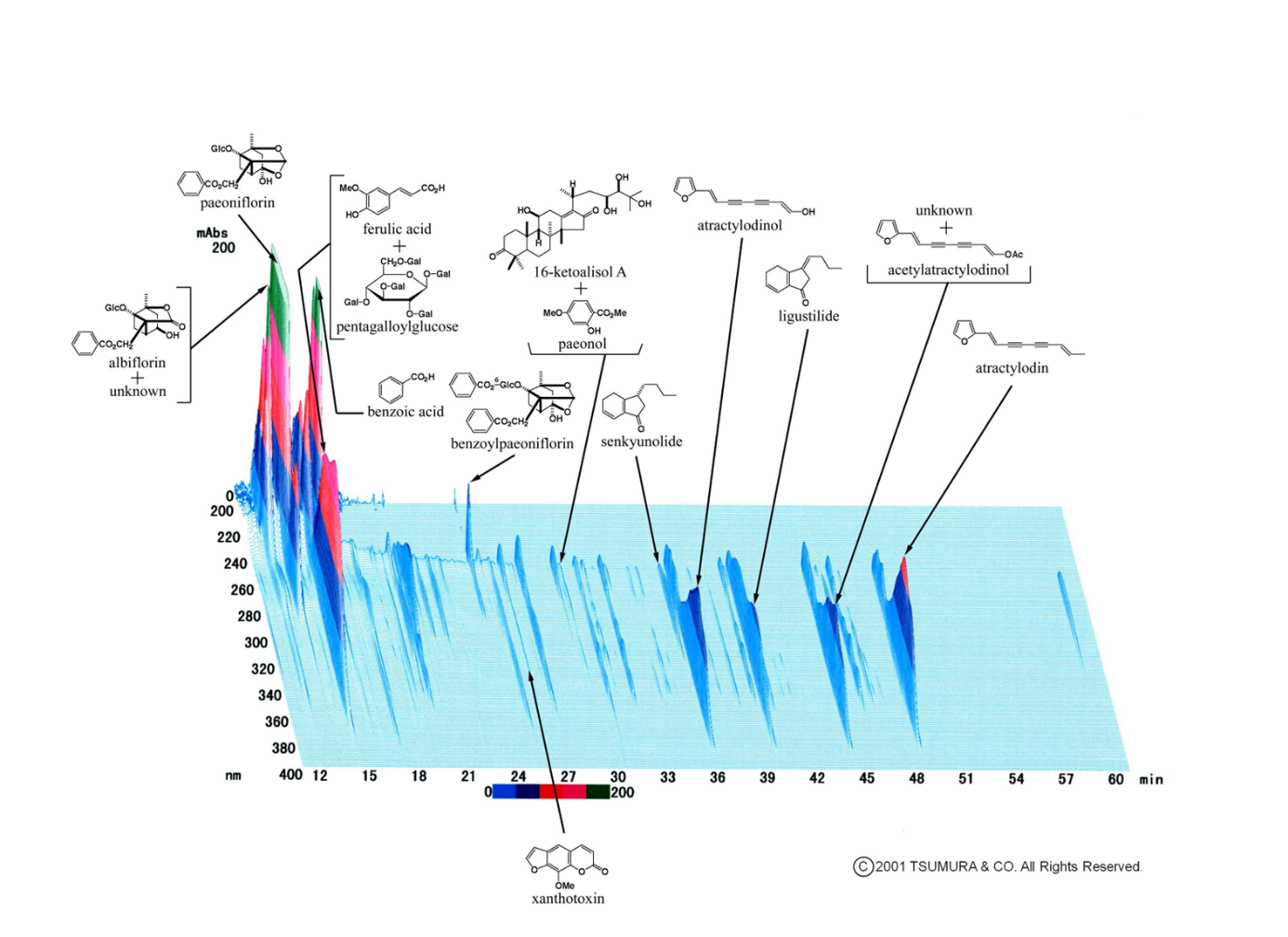


**Supplementary Figure 1.** A three-dimensional high-performance liquid chromatography profile of tokishakuyakusan, provided by TSUMURA & CO., Tokyo, Japan.


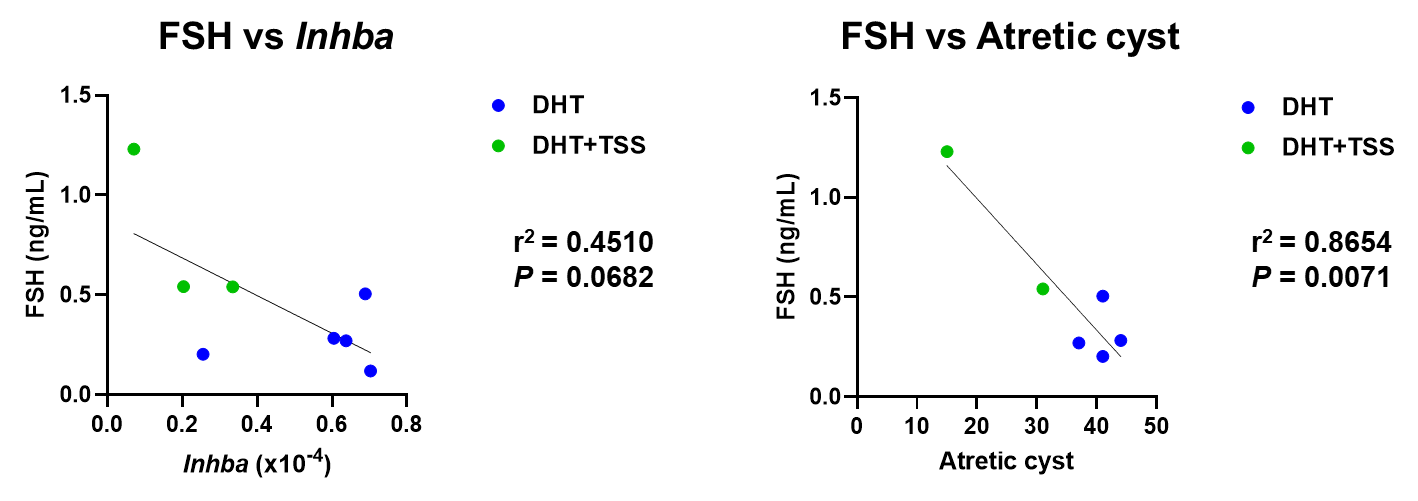


**Supplementary Figure 2.** Correlation analysis between detectable serum FSH levels, *inhba* expression, and ovarian follicle counts in DHT and DHT+TSS groups. The correlation coefficient (r^2^) and *P*-value were calculated. DHT, 5α-dihydrotestosterone; TSS, tokishakuyakusan; FSH, follicle-stimulating hormone; Inhba, inhibin-βa.


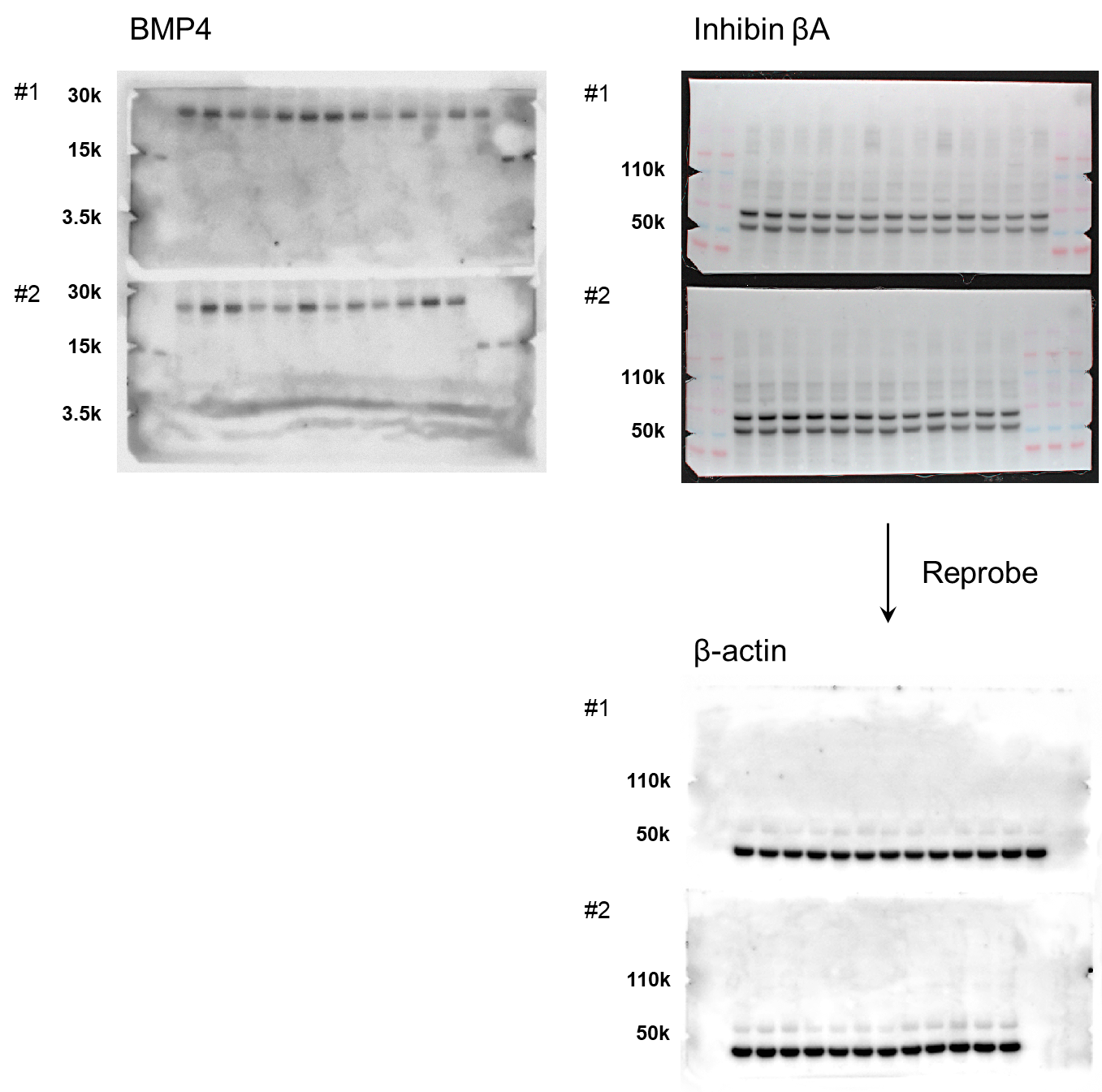


**Supplementary Figure 3.** Western Blot images of BMP4, Inhibin βA, and β-actin proteins. BMP, bone morphogenetic protein


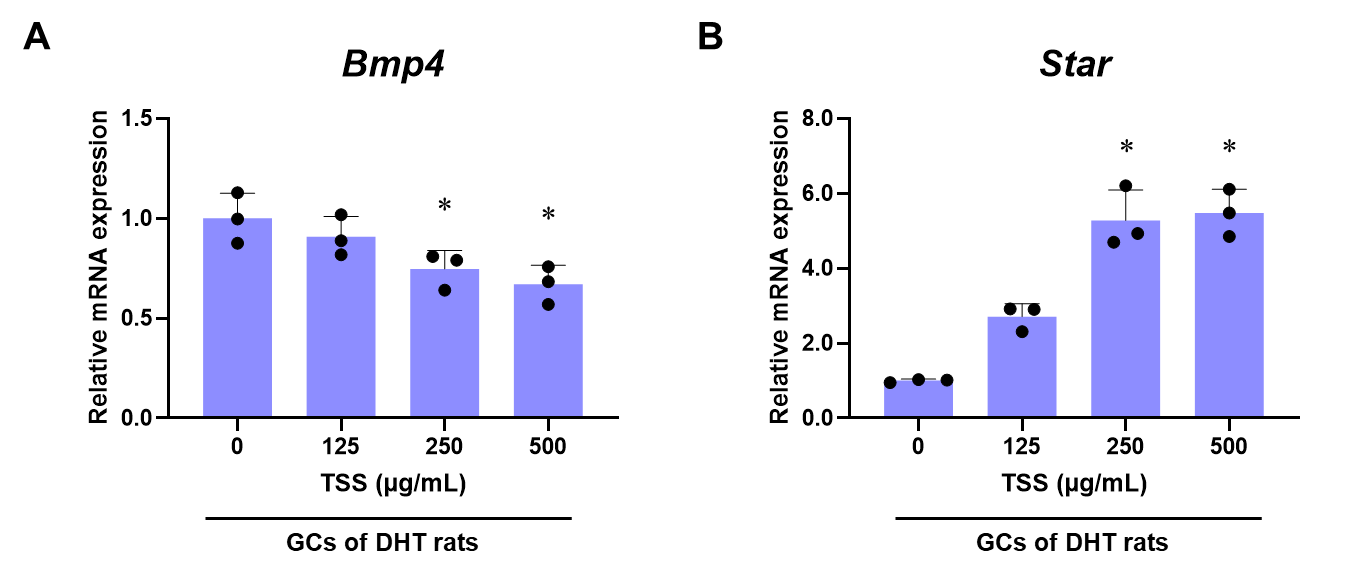


**Supplementary Figure 4.** Effect of tokishakuyakusan (TSS) on *Bmp4* and *Star* expressions in granulosa cells (GCs) derived from prenatally 5α-dihydrotestosterone (DHT)-treated rats. (A, B) Relative mRNA expression of *Bmp4* and *Star* in primary cultured GCs after 24 h of TSS treatment (125–500 μg/mL) with FSH (3 ng/mL). Expression levels are relative to *Gapdh*. Data are shown as mean ± standard deviation with individual data points (n=3 per group). **P* < 0.05, *P*-values for the TSS-untreated control were obtained using One-way analysis of variance (ANOVA) followed by Dunnett’s post-hoc test (*Bmp4*) or Kruskal–Wallis test followed by Dunn's test (*Star*). FSH, follicle-stimulating hormone; Bmp, bone morphogenetic protein; Star, steroidogenic acute regulatory protein; Gapdh, glyceraldehyde-3-phosphate dehydrogenase.


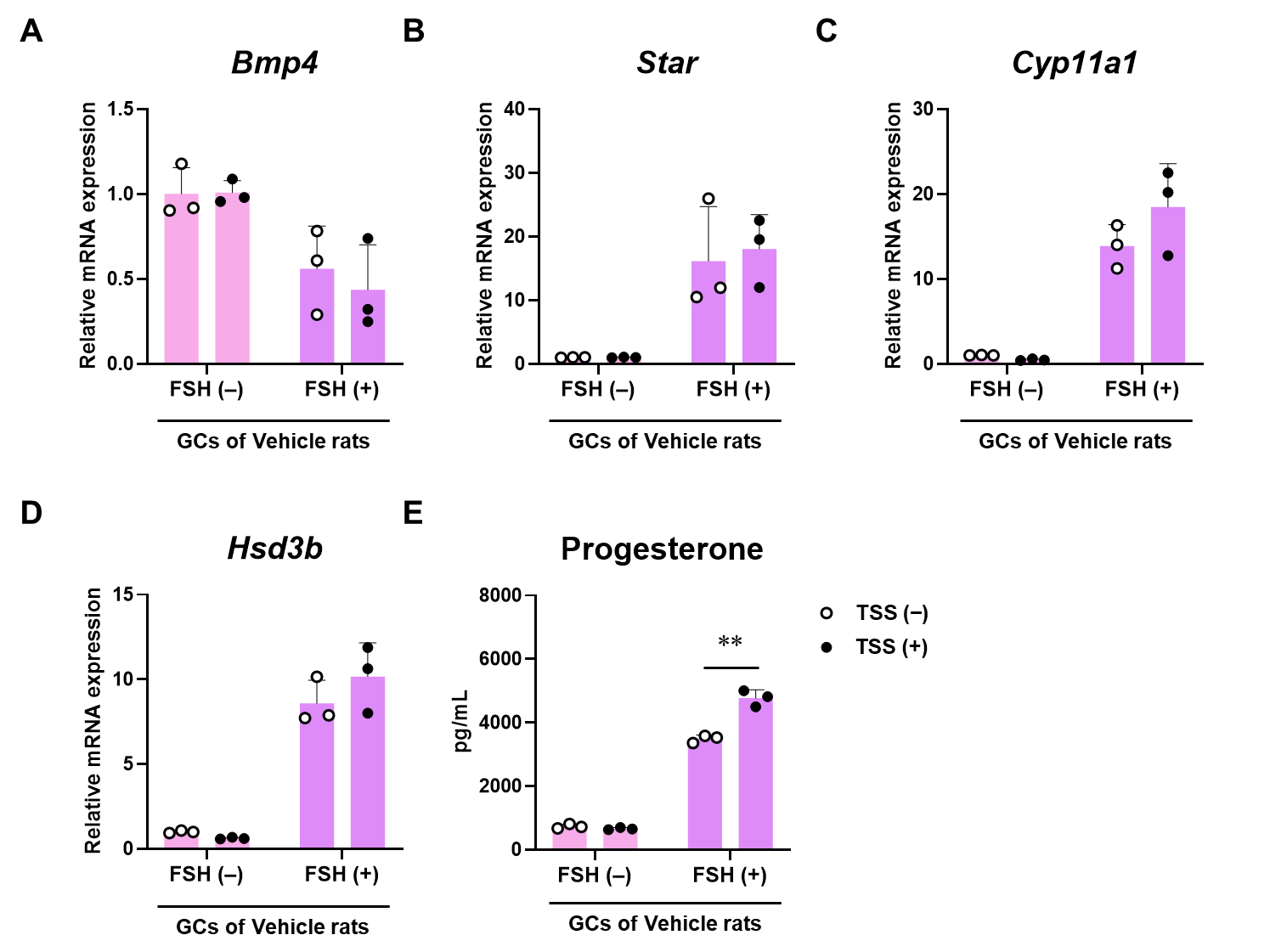


**Supplementary Figure 5.** Effect of tokishakuyakusan (TSS) on progesterone synthesis in granulosa cells (GCs) derived from prenatally vehicle-treated rats. (A-D) Relative mRNA expression of *Bmp4* (A), *Star* (B), *Cyp11a1* (C), and *Hsd3b* (D) in primary cultured GCs after 24 h of TSS (500 μg/mL) treatment with or without FSH (3 ng/mL). Expression levels are relative to Gapdh. (E) Progesterone concentration in GC culture medium after 24 h of treatment. Data are shown as mean ± standard deviation with individual data points (n=3 per group). **P < 0.01, two-way analysis of variance (ANOVA) followed by Tukey’s post-hoc test. FSH, follicle-stimulating hormone; Star, steroidogenic acute regulatory protein; Cyp11a1, cytochrome P450 11A1; Hsd3b, 3β hydroxysteroid dehydrogenase; Gapdh, glyceraldehyde-3-phosphate dehydrogenase.

## Supplementary Table

**Supplementary Table 1.** Gene expression profiling using TaqMan Array plates
